# Structure and interstitial iodide migration in hybrid perovskite methylammonium lead iodide

**DOI:** 10.1038/ncomms15152

**Published:** 2017-05-11

**Authors:** J. L. Minns, P. Zajdel, D. Chernyshov, W. van Beek, M. A. Green

**Affiliations:** 1 School of Physical Sciences, Ingram Building, University of Kent, Canterbury, Kent CT2 7NH, UK; 2 Institute of Physics, University of Silesia, ul. Uniwersytecka 4, 40007 Katowice, Poland; 3 Swiss-Norwegian Beam Lines, European Synchrotron Radiation Facility, Polygone Scientifique Louis Néel, 6, Rue Jules Horowitz, 38000 Grenoble, France

## Abstract

Hybrid perovskites form an emerging family of exceptional light harvesting compounds. However, the mechanism underpinning their photovoltaic effect is still far from understood, which is impeded by a lack of clarity on their structures. Here we show that iodide ions in the methylammonium lead iodide migrate via interstitial sites at temperatures above 280 K. This coincides with temperature dependent static distortions resulting in pseudocubic local symmetry. Based on bond distance analysis, the migrating and distorted iodines are at lengths consistent with the formation of I_2_ molecules, suggesting a 2I^−^→I_2_+2*e*^−^ redox couple. The actual formula of this compound is thus (CH_3_NH_3_)PbI_3−2*x*_(I_2_)_*x*_ where *x*∼0.007 at room temperature. A crucial feature of the tetragonal structure is that the methylammonium ions do not sit centrally in the A-site cavity, but disordered around two off-centre orientations that facilitate the interstitial ion migration via a gate opening mechanism.

## Abstract (web summary)

The mechanism underpinning the photovoltaic effect in hybrid perovskite solar cells has remained unclear. Here, Green and co-workers suggest that iodide ions in methylammonium lead iodide perovskite migrate via interstitial sites and undergo a redox reaction to form molecular iodine and free electrons.

Perovskite structures, with the general formula, ABO_3_, form one of the most important and commercially exploited family of solids. Hybrid perovskites that contain both organic and inorganic components are a subset, where the A site is composed of an organic cation, such as methylammonium (MA), within a post transition metal halide framework, such as lead iodide. They have emerged since 2009 (ref. 1) as simple, low cost solar cell materials, with power conversion efficiencies that are becoming competitive with silicon^2^,^3^,^4^,^5^,^6^,^7^. Methylammonium lead iodide (MAPbI) undergoes a number of structural phase transitions as a function of temperature, including an orthorhombic—tetragonal—cubic evolution that is common in perovskites. Ion mobility and the rotational dynamics of the non-isotropic MA ion adds further complexity to the structure. Mobility of all three ions, Pb, I and MA, have been extensively studied^8^,^9^,^10^,^11^,^12^, but, as yet, no definitive mechanism has emerged, although iodide ions have been shown to play a key role^13^. The complex structural features and intrinsic disorder explains the large number of anomalies in the literature as to the exact symmetry and structural parameters^14^, which is suggestive of localized symmetry variations that is prevalent in solid electrolytes.

We show that iodide ions migrate through an interstitial (I3) position. This migration is only possible through a correlated rearrangement of the MA ions. Furthermore, a substantial local static distortion of the Pb–I octahedra into a pseudocubic arrangement produces I–I bond distances consistent with the formation neutral I_2_ defects that could effectively act as electron/hole pairs.

## Results

### X-ray and neutron diffraction

The maximum entropy method (MEM) is an analysis technique that can be applied to diffraction data that generates density maps without prior knowledge of symmetry and unit cell content, and therefore unbiased towards any specific structural model. It can provide information on subtle local distortions even when this scattering is extremely weak compared to the bulk diffraction^15^,^16^,^17^.

**Figure 1:**
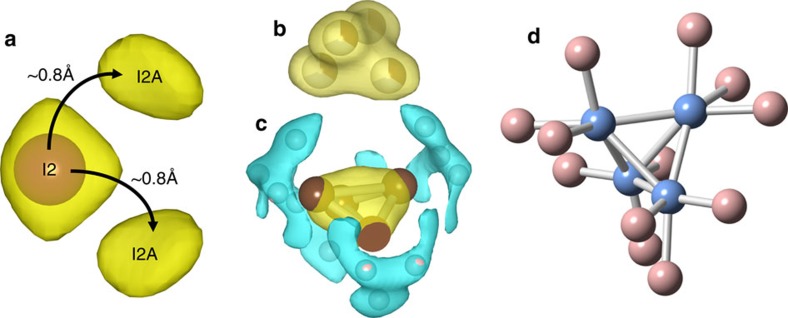
Key structural features of MAPbI obtained from MEM analysis. (**a**) Section of the (100) projection of the nuclear scattering density (yellow) at room temperature (isosurface level of 0.8 fmÅ^−3^) showing main iodide position (I2, purple sphere) is accompanied by two additional scattering densities (labelled I2A). (**b**) X-ray scattering and (**c**) nuclear scattering density map of methylammonium molecules (isosurface level of 1.0 fmÅ^−3^), showing C and N (yellow) and hydrogen scattering (blue) (**d**) molecular structure extracted from the maxima in the scattering density maps.

To gain a deeper insight into the structure of MAPbI we have performed both powder neutron, single crystal X-ray diffraction, and powder synchrotron X-ray studies. Details of the structure determination strategy are given in the Supplementary Note 1. The structure was solved in *I4/m* space group with lattice parameters of *a=*8.8756(1) Å and *c*=12.6517(3) Å. The *I4/m* symmetry is not an isomorphic subgroup of the high temperature cubic perovskite 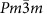 space group, so not a common perovskite symmetry^18^. However, in the case of MAPbI the transition from cubic to tetragonal is first order, so multiple irreducible representations can be adopted. The principal features of the MAPbI structure derived from the MEM analysis was found to be considerably more complex than previous realized (Fig. 1). Supplementary Fig. 1 shows the crystal structure from positions extracted from MEM analysis of powder neutron diffraction data (Supplementary Fig. 2). The nuclear scattering density around the iodide ion at the (−0.2148(3) −0.2851(3) 0.5) position (I2) at room temperature was found to be localized with typical levels of thermal distribution (Fig. 1a). However, additional densities with similarly localized scattering were identified at two positions in close proximity, demonstrating static disorder of the I2 site, labelled I2A. These were determined to be at (−0.252(3) −0.248(3) 0.453) in a pseudocubic arrangement, and represent a ∼0.8 Å shift from the known I2 position towards the MA ions and lying on either side of a mirror plane in the tetragonal space group. Supplementary Fig. 3 shows how the local tilting of the perovskite is different in I2A and I2 positions. The MEM density maps surrounding the MA ion for single crystal X-ray and powder neutron diffraction showed similar scattering for the C–N that is best described as a 4 atom tetrahedron unit, with the centre of the tetrahedron in the middle of the A-site cavity (Fig. 1b,c, respectively). In addition to the scattering for C/N, the neutron diffraction data showed scattering from 6 hydrogen positions just over 1 Å from the C/N positions, with considerable amount of disorder through libration, as well as rotational disorder, which is consistent with inelastic neutron scattering results^19^,^20^. A model was derived for the MA ion (Fig. 1d), where there are two orientations for the CH_3_NH_3_ molecule, one in the (220) plane and one in the 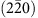 plane, similar to the arrangement found from single crystal neutron diffraction^21^. These orientations point exactly between the iodide positions, I2, in the *z*=0.25 plane. However, whereas the centre of mass of the two molecule orientations lie also at *z*=0.25, the two molecules were found to be at off-centre positions of *z*=0.221(3) and 0.279(3). The orientation of the MA molecule is an important component of the structure as it has been found to greatly influence the electronic properties of MAPbI^22^.

**Figure 2:**
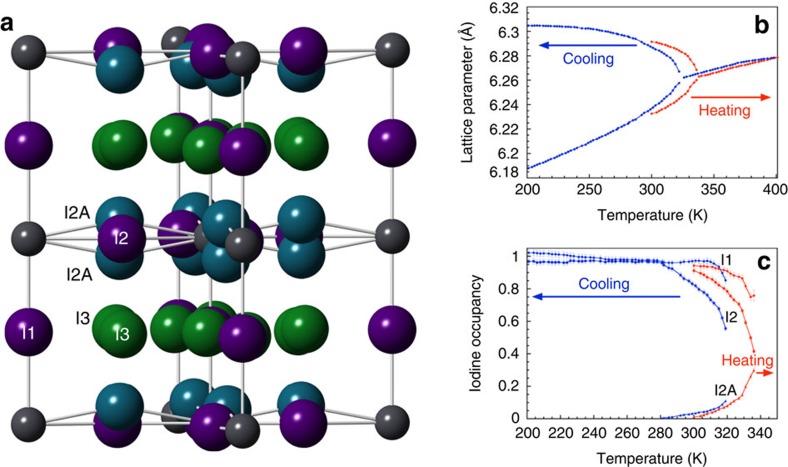
Structure of MAPbI and variation of occupancy and lattice parameter with temperature. (**a**) Lead (grey spheres) and iodide positions in the room temperature *I4/m* space group, showing four crystallographically inequivalent iodide positions within the unit cell at position I1 and I2 (purple) that form the regular perovskite PbI_6_ corner shared octahedra and two additional position I2A (blue sphere) and I3 (green sphere). The methylammonium ions are omitted for clarity. Powder synchrotron X-ray data shows (**b**) hysteresis in tetragonal to cubic phase transition, and (**c**) temperature dependence of the I1, I2 and I2A iodide ion site occupancy as a function of temperature.

A further iodide position (I3) was observed in the powder neutron diffraction, powder synchrotron diffraction and single crystal X-ray diffraction that sits in an interstitial site in the z∼0.25 plane with Pb and MA ions (Fig. 2a). Determination of the evolution of structure and composition as a function of temperature (Fig. 2b) showed a hysteresis effect in the heating and cooling profiles, demonstrating the phase transition is first order. Supplementary Fig. 4 shows the temperature dependence of specific reflection as measured from synchrotron radiation. The lattice parameters dependence showed a tetragonal to cubic transition at ∼335 K, whereas this was suppressed to ∼320 K on cooling (Fig. 2b). This 15 K variation is mirrored in the composition variation (Fig. 2c) where the I1, I2 and I2A sites were similarly shifted. There was a slight drop in the composition of I1 sites close to the transition to the cubic phase, but the largest variation was in the occupancies of the I2 and I2A ions, where a substantial drop in I2 composition was observed with increases in I2A, but not to the same extent. The I3 content was difficult to accurately determine with the short runs of the synchrotron measurements. However, the total composition was seen to drop slightly, implying the I3 site were being populated, but were diffuse and thereby not contributing to the Bragg scattering to the same extent as the other iodide ions.

### Ion migration mechanism

**Figure 3:**
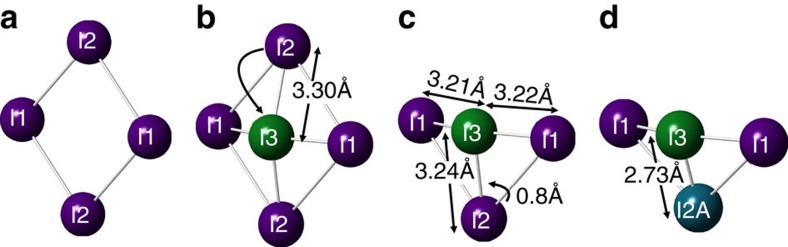
Proposed mechanism for molecular iodine formation in MAPbI (**a**) Section of the perovskite structure showing two I1 and two I2 position lying in the 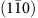 plane, (**b**) Iodine I2 moves to the interstitial I3 position leaving (**c**) I3 surrounded by three roughly equidistant iodine ions, provoking I2 ions to jump to a I2A position creating (**d**) bond formation to produce I_2_ molecules.

**Figure 4:**
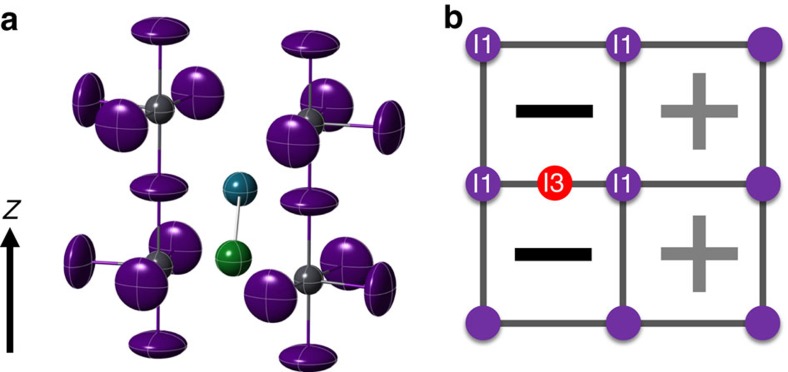
Representative local structure of MAPbI after the proposed diatomic iodine formation. (**a**) Relative positions of I_2_ molecule (green and blue sphere) compared to the perovskite framework. Methylammonium ions are omitted for clarity, and (**b**) cooperative arrangements of the orientation of the methylammonium ions as a result of occupation of I3 sites. (−) and (+) represents single and shared orientations of MA molecule, respectively.

From the isolation of these atomic positions and variation of compositions, one can propose a mechanism for ion migration within the cell. The significant drop in composition of the I2 site at temperatures above 280 K, compared with the increased occupancy of the I2A site, implies that I2 are both populating the ion interstitial site, I3, and well as shifting to the new I2A locations. A I2 to I3 hop (Fig. 3a–c) would leave the I3 ions surrounded by three iodide ions all approximately 3.2 Å apart. Given the concomitant increase in the population of the both the I2A and I3 sites over the same temperature regime, the nature of the bonding between these two positions is important. Supplementary Fig. 5 shows the bond distances when all four iodide positions are populated. Polyiodide ions are well known to the form multiple low valent iodide chains, where the I–I bond lengths are extremely sensitive to the nature of the bonding and charges on the iodine^23^. Structure of solid I_2_ is an orthorhombic zig-zag structure with intramolecular I–I bond lengths of 2.68 Å, and intermolecular I_2_ distances of 3.56 Å (ref. 24). This value of 2.68 Å can be considered as a primary covalent I–I bond and other forms of iodine chains where I_2_ donates to the σ* antibonding orbital in a charge transfer complex have significantly larger bond lengths^23^,^25^. For example, the triiodide ion, [I_3_]^−^ in orthorhombic CsI_3_ has I–I bond lengths of 2.84 Å and 3.04 Å (ref. 26); one slightly higher than seen in covalent I_2_ and one longer bond possessing most of the additional charge. I_2_ confined within frameworks have similar bond lengths, such as iodine in formate, Zn_3_(HCOO)_6_, has a bond length of 2.691 Å with a second weakly interacting molecule at 3.59 Å (ref. 27). In contrast, the [I_2_]^+^ ion in I_2_Sb_2_F_11_ has a shorter I–I bond length of just 2.56 Å (ref. 28). The two I3–I2A bond lengths in MAPbI are at 2.7(1) Å and 2.6(1) Å, so from these structural considerations it is consistent with the static disorder and shift from I2 to I2A is the result of covalent I_2_ bond formation in MAPbI to produce a neutral diatomic I_2_ molecule within the perovskite framework. This would have extensive implications on the band structure and charge transfer suggests a redox reaction of 2I^−^→I_2_+2*e*^−^. The resulting structure (Fig. 4a) shows potential chains of I_2_ and I^−^ ions along the *z* axis. However, the bond distances between I3 and both orientations of the MA ions are unphysical, which suggests that the occupancy of I3 can only be achieved with the MA molecule adopting a perpendicular orientation (Fig. 4b), such that diffusion of the I3 ions from I2 and I1 only occurs with collective motion of the MA ions in a gate opening type mechanism. Further evidence of the dynamical hybrid structure was provided by Raman spectroscopy, which is shown in Supplementary Fig. 6, and Supplementary Note 2. All final derived atomic coordinates, occupancies are provided in Supplementary Tables 1–4.

Iodine, I_2_, itself is a semiconductor^29^, although can be highly conducting in polyiodide structures, or as inclusion in other frameworks creating charge transfer complexes^23^. A number of metal-organic frameworks materials have shown considerable change to their electronic structure and electrical conductivity with the inclusion of iodine^30^,^31^. Typical dye-sensitized solar cells require an additional TiO_2_ layer to act as an electron acceptor. However, it has been established that the hybrid perovskite itself acts as a free electron carrier without the need for complex nanostructures^3^,^5^,^32^, although the underlying mechanism for such characteristics is not clear. Further studies will be needed to clarify the presence and role of the I_2_/I^−^ redox couple and the implied electron/hole formation within hybrid perovskite, as well as the effect on the electronic and ionic conduction and whether this is related to its solar conversion properties, such as the long electron-hole diffusion lengths^33^ and lifetimes^34^, and photon recycling^35^.

## Methods

### Synthesis and data treatment

Crystals of MAPbI with a volume of ∼2 cm^3^ were grown by slow evaporation of CH_3_NH_3_I and PbI_2_ in γ-butyrolactone over a 14-day period. These crystals were ground to perform the powder neutron and X-ray studies, and smaller crystals were cleaved to perform the single crystal X-ray studies. Powder neutron diffraction data were collected on the BT1 diffractometer at NCNR at the National Institute of Standards and Technology, Gaithersburg, MD, USA using Cu(311) monochromator (*λ*=1.5401(1) Å). Single crystal X-ray diffraction data was collected on a dual-source Rigaku Oxford Diffraction Supernova diffractometer. Powder synchrotron diffraction experiments were performed at the Swiss-Norwegian beamline (SNBL) at the ESRF, France, with an incident wavelength of *λ*=0.6932 Å. Whole pattern fitting based on MEM was carried out using PRIMA^36^ and RIETAN^37^ with a 96 × 96 × 128-voxel density map for both the powder neutron and single crystal X-ray diffraction data. MEM density maps were analysed using the Vesta program^38^. The FULLPROF^39^ suite of programs were used to perform Rietveld refinement on both the neutron and synchrotron X-ray powder diffraction data. Refinement of single crystal X-ray diffraction data were performed using the SHELX program^40^. Raman spectroscopy was performed with a Horiba LabRAM S3000 Raman microscope using a near infrared excitation wavelength of 784.15 nm, and described in more detail in Supplementary Methods.

### Data availability

The data that support the findings of this study are available from the corresponding authors upon reasonable request. The X-ray crystallographic coordinates for the structure reported in this study have been deposited at the Cambridge Crystallographic Data Centre (CCDC), under deposition number 1535723. These data can be obtained free of charge from The Cambridge Crystallographic Data Centre via  www.ccdc.cam.ac.uk/data_request/cif.

## Additional information

**How to cite this article:** Minns, J. L. *et al*. Structure and interstitial iodide migration in hybrid perovskite methylammonium lead iodide. *Nat. Commun.* **8,** 15152 doi: 10.1038/ncomms15152 (2017).

**Publisher's note**: Springer Nature remains neutral with regard to jurisdictional claims in published maps and institutional affiliations.

## Supplementary Material

Supplementary Information Supplementary Figures, Supplementary Tables, Supplementary Notes and Supplementary References
