# Supplementary material for: Structure and interstitial iodide migration in hybrid perovskite methylammonium lead iodide
Source: Nat Commun. 2017 May 11;8:15152. doi: 10.1038/ncomms15152 (PMC5437276; doi:10.1038/ncomms15152)
Supplement: Supplementary Information — Supplementary Figures, Supplementary Tables, Supplementary Notes and Supplementary References [file ncomms15152-s1.pdf]

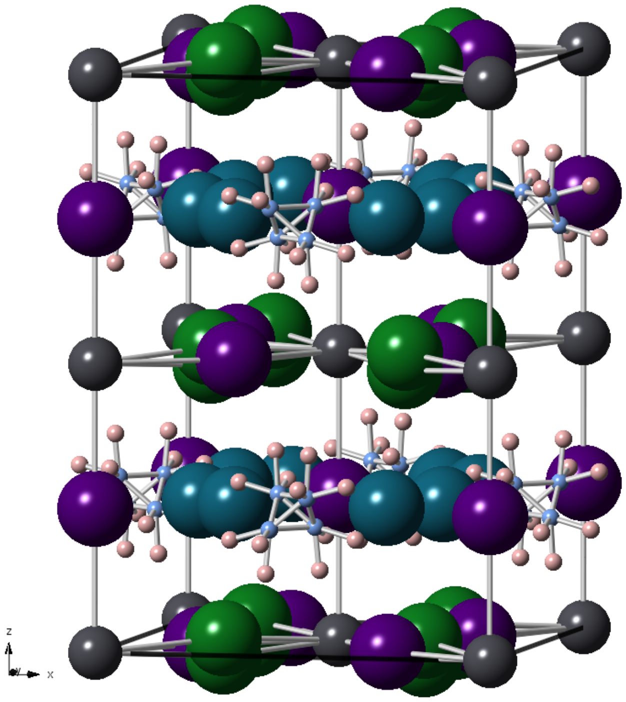

Supplementary Figure 1. Crystal structure of MAPbI under ambient conditions. Estimated atomic coordinates were extracted from maxima in the nuclear scattering density reconstructed from powder neutron diffraction data. Atoms shown are lead (grey), iodine (I1 and I2 (purple), I2A (green) and I3(blue)), C/N (sky blue) and H (pink).

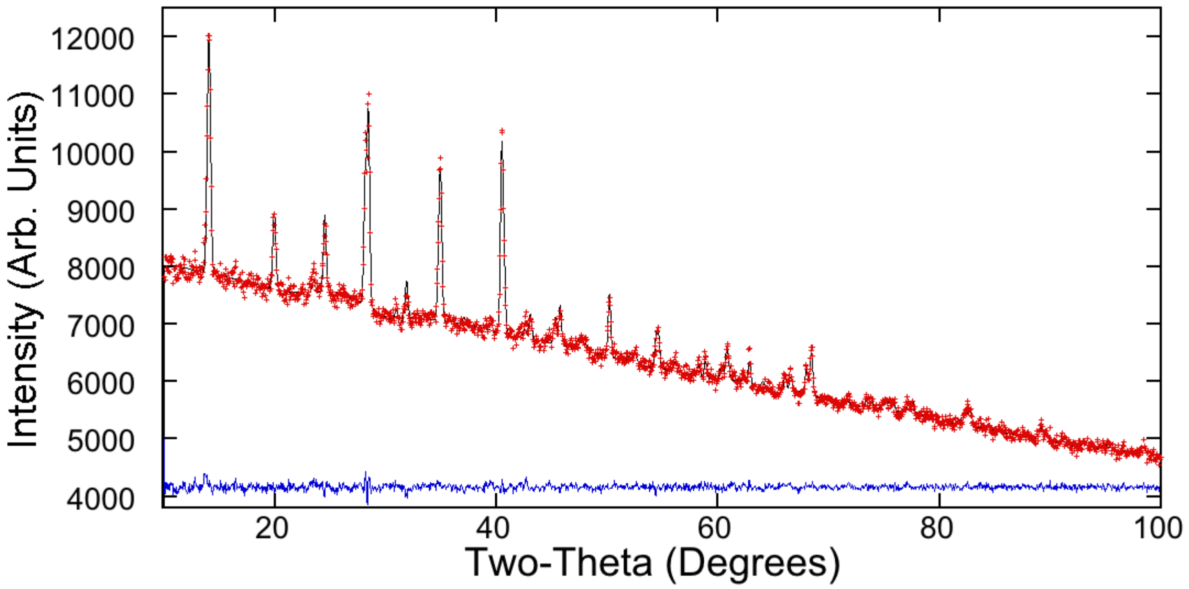

Supplementary Figure 2. Rietveld refinement of powder neutron diffraction data of MAPbI under ambient condition. The observed (red), calculated (black) and difference (blue) intensities are shown as a function of two-theta.

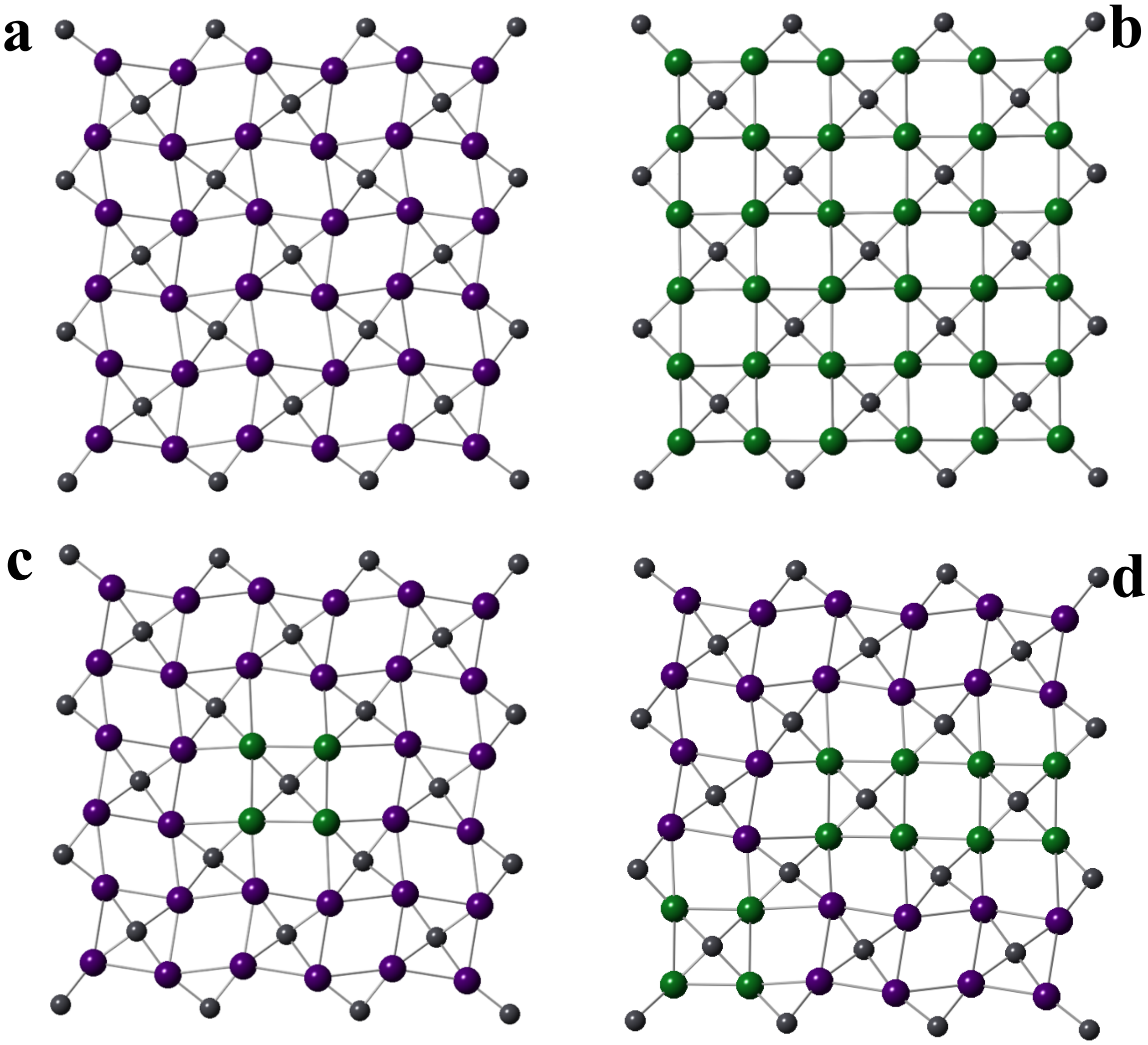

Supplementary Figure 3. Local compared with bulk atomic structure of MAPbI under ambient conditions showing the [001] slice with a, only I1 and I2 ions (purple) and **b**, hypothetical structure with only pseudocubic I2A ions (green) and **c**, representation of low temperature structure with a mixture of I1, I2 and I2A ions demonstrating how the I2A ions provide different local symmetry of the PbI_6 octahedron and **d**, representation of the structure at higher temperatures with an increase in the presence of I2A ions compared with **c**. Pb is shown in grey.

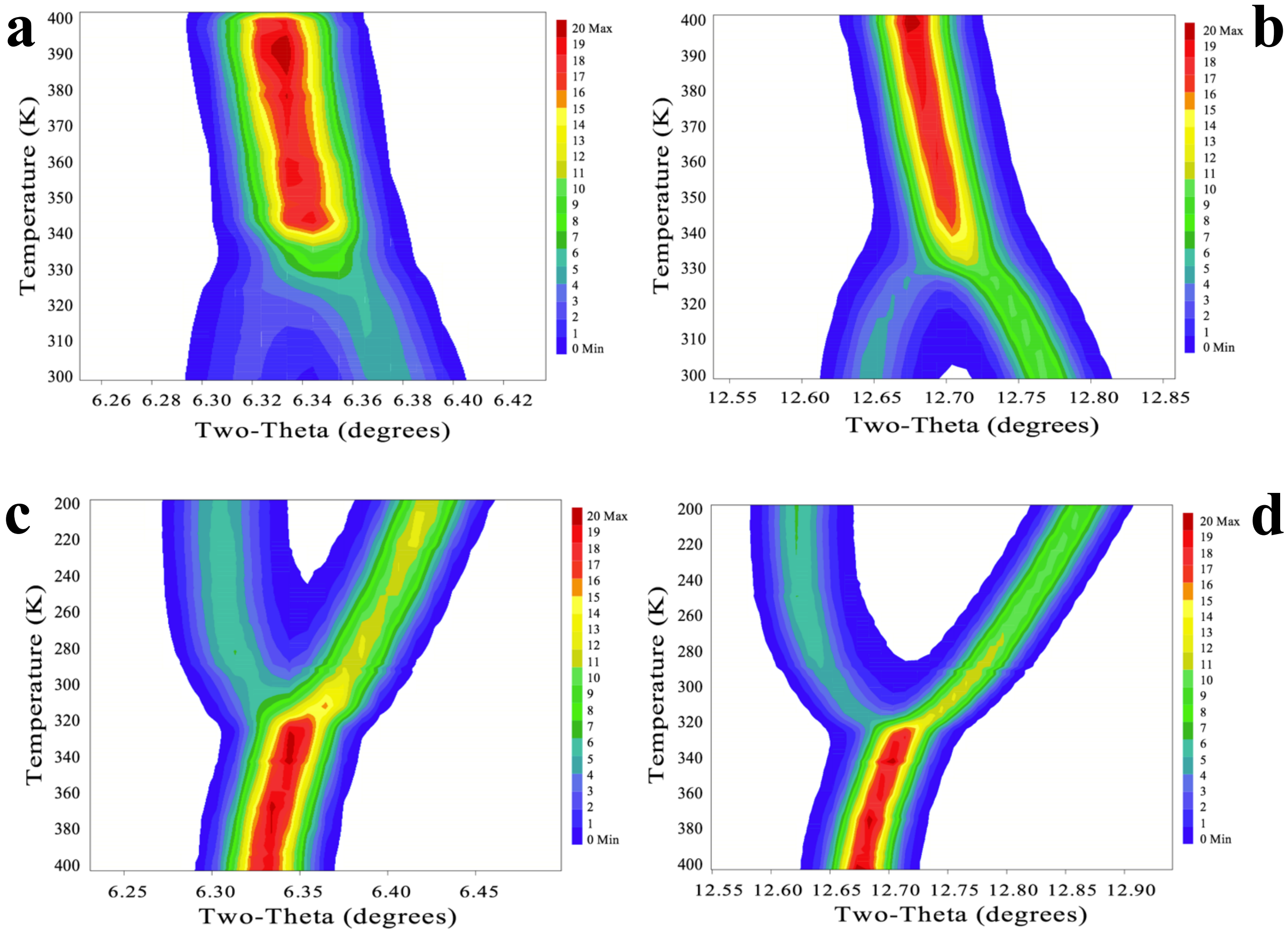

Supplementary Figure 4. Temperature dependence of the synchrotron X-ray diffraction data of MAPbI showing evolution of the a, tetragonal (002) and (110) reflections to the cubic (200) reflection at around 6.3º and **b,** tetragonal (004) and (220) reflections to the cubic (400) over the temperature ranhe of 300 to 400 K. Panel **c** and **d** show the reverse transition from 400 to 200 K.

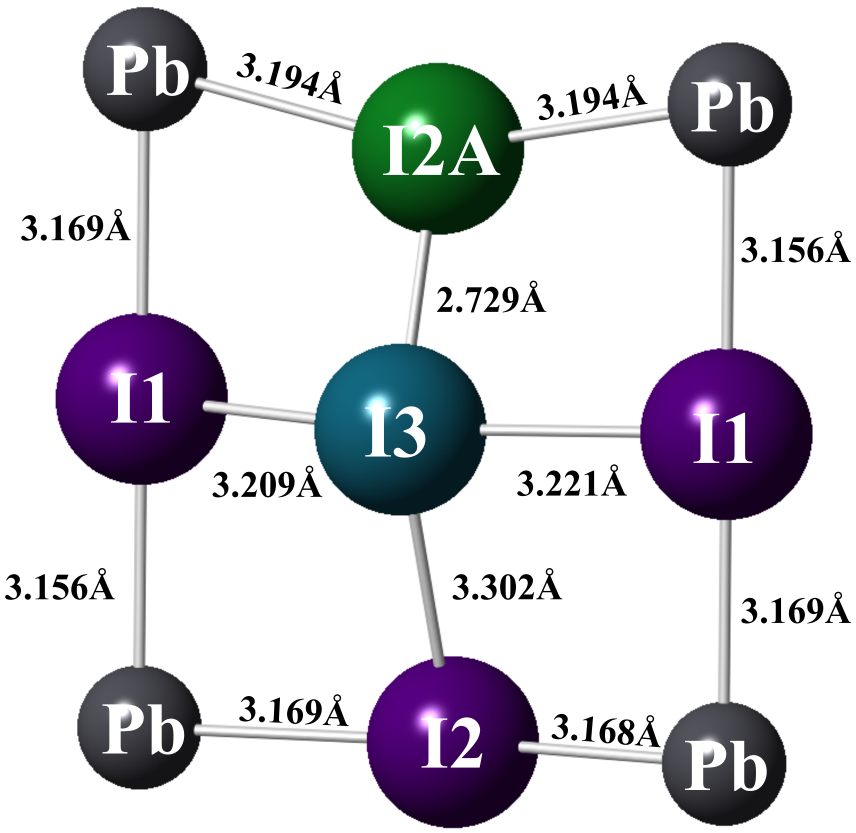

Supplementart Figure 5. Pb – I environment in MAPbI with inclusion of I3 interstitial iodide ion. A temperature dependent proportion of the I2 ions (purple) show a significant shift to I2A (green) yielding a I2A – I3 (blue) bond of around 2.7Å, consistent with the formation of I_2 and polyiodide structures. Pb is shown in grey.

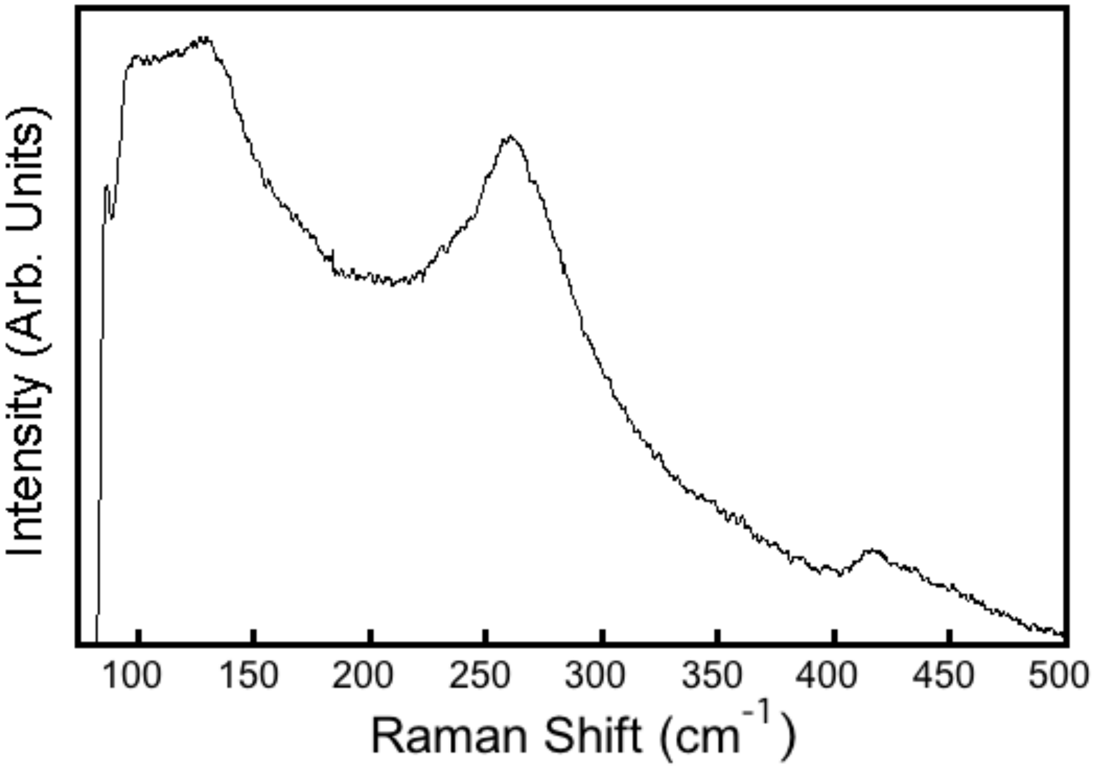

Supplementary Figure 6. Raman spectra of MAPbI at ambient condition.

Supplementary Table 1. Estimated atomic coordinates of MAPbI under ambient conditions extracted from maxima in the reconstructed nuclear scattering density maps using powder neutron diffraction.

Atom	*x*	*y*	*z*
Pb1	0.5	0.5	0.5
Pb2	0.5	0.5	0.0
I1	0.5	0.5	0.75
I2	0.213475	0.286460	0.500000
I2A	0.302085	0.197915	0.554690
I3	-0.191000	-0.311000	0.251000
C1/N1	0.546600	-0.046600	0.277600
H1	0.146400	0.546400	0.700000
H2	-0.030600	0.353500	0.292300
H3	0.445300	-0.050400	0.851000

Supplementary Table 2. Structure of MAPbI under ambient conditions. Atomic coordinates of MAPbI obtained from Rietveld refinement of powder neutron diffraction data. Cell parameters were refined to be *a,b* = 8.8618(4) Å and c = 12.6588(8) Å with a volume of 994.11(9) Å.

Atom	*x*	*y*	*z*	U_{iso} (Å^3)	Occupancy
Pb1	0.5	0.5	0.5	0.036(2)	
Pb2	0.5	0.5	0.0	0.036(2)	
I1	0.5	0.5	0.75(3)	0.092(6)	0.92(3)
I2	0.21(4)	0.29(4)	0.5	0.092(6)	0.90(4)
I2A	0.27(7)	0.24(7)	0.449(3)	0.092(6)	0.06(2)
I3	-0.21(4)	-0.31(4)	0.25(4)	0.092(6)	0.004(3)
C1/N1	0.55(1)	-0.04(1)	0.278(6)	0.25(4)	
H1	0.15(2)	0.55(2)	0.70(1)	0.089(7)	
H2	-0.03(2)	0.35(2)	0.29(1)	0.089(7)	
H3	0.44(3)	-0.05(3)	0.851(3)	0.089(7)	

Supplementary Table 3. Structure of MAPbI under ambient conditions.
Crystallographic parameters and experimental condition obtained from single crystal X-ray diffraction.

Chemical formula	Pb I3 C N H6
Molar mass, SG, Z, T(K)	619.98, I 4/m (#87), 4, 300.0(1)
a, b, c (Å) *α, β, γ* (°), *V* (Å^3)	8.87560(10), 8.87560(10), 12.6517(3), 90, 90, 90, 996.65(3)
Radiation source	SuperNova (Mo) X-ray Source, mirror, MoKα, 0.71073
Density(g cm^{-3}), μ (mm^{-1})	4.132, 26.137
Crystal form, colour, dimensions	Multifaceted, dark, 0.13 (max), 0.045 (min), 0.079 (mid)
Diffractometer	Four-circle diffractometer SuperNova, Dual, Cu, AtlasS2
Data collection method	ω scans
Absorption correction	CrysAlisPro 1.171.38.41 (Rigaku OD, 2015) Numerical absorption correction based on gaussian integration over a multifaceted crystal model. T_{min} *0.093,* T_{max} *0.391* Empirical absorption correction using spherical harmonics, implemented in SCALE3 ABSPACK scaling algorithm.
# reflections: measured, independent, observed	10920, 947, 801
Criterion for observed	I > 2σ(I)
R_{int}, R_{sigma}, θ_{min} (°), θ_{max} (°), θ_{full} (°)	3.74, 4.58, 3.22, 32.91 (meas. frac. 96.9 %), 25.24 (meas. frac. 99.8 %)
Software	SHELXS, SHELX (2013/14)
R1[F^2 > 2σ(F^2)], R_{all}, *wR*(F^2), *S*, No. reflections, parameters	3.92, 4.62, 9.93, 1.400, 947, 32
Weighting scheme	(SHELXL) w = 1/[2 (F^2_o) + (0.0 P)^2 + 40.8 P], where P = max(F^2_o + 2 F^2_c)/3
$(\Delta/\sigma)_{max}$, $\Delta\rho_{max}$, $\Delta\rho_{min}$ (e Å^{-3})	0.022, 1.50, -1.80
Extinction correction	SHELXL, 0.00070(6)

Single Crystal X-Ray Diffraction Atomic Coordinates

Atom	x	y	z	U(Å^3)	Occupancy
Pb1	0	0	0	0.0302(3)	1
Pb2	0	0	0.5	0.0302(3)	1
I1	0	0	0.24949(18)	0.0851(11)	0.971(9)
I2	-0.2148(3)	-0.2851(3)	0.5	0.0776(11)	0.925(13)
I2A	-0.252(3)	-0.248(3)	0.453(2)	0.045(7)	0.042(6)
I3	0.194(11)	0.305(11)	0.245(9)	0.04(3)	0.007(3)
C1/N1	-0.035(8)	0.438(6)	0.221(3)	0.17(3)	0.36(2)

Supplementary Table 4. Structure of MAPbI under ambient conditions.
Crystallographic parameters obtained from synchrotron powder X-ray diffraction.

Atom	x	y	z	B(Å^3)	Occupancy
Pb1	0	0	0	0.2(2)	1
Pb2	0	0	0.5	0.2(2)	1
I1	0	0	0.251(1)	3.3(3)	0.971(9)
I2	0.285(2)	0.215(2)	0.0	3.3(3)	0.91(2)
I2A	0.248(3)	0.252(3)	0.047(2)	3.3(3)	0.03(1)
I3	0.306(3)	0.194(3)	0.251(3)	3.3(3)	0.004(2)
C1/N1	0.452(3)	0.048(3)	0.220(3)	28(1)	1

Supplementary Note 1

To perform the structure determination a combination of three techniques were employed; single crystal X-ray diffraction, powder neutron diffraction and synchrotron X-ray diffraction. In addition, the data was analyzed by both reconstruction of the electron (X-ray) and nuclear (neutron) scattering density by the maximum entropy method (MEM), and conventional atomic coordinate refinement procedures. A summary of the strategy is provided below:

1. Powder neutron diffraction data were collected on the BT1 diffractometer at the NCNR, NIST over a 3-day period. Maximum entropy method analysis was used to reconstruct the nuclear scattering density maps. Maxima in the nuclear density were extracted to estimate atomic positions. Although it should be noted that some areas of these densities are not localized into specific atomic positions, as a result of considerable amount of disorder from the methylammonium ions, as well as migrating iodide ions. Therefore, for certain atoms, such as the hydrogen, atomic coordinates do not give an accurate description of the overall nuclear density. Specific localized coordinates were derived for I2 as well as providing evidence for a static distortion within the structure from I2 to I2A positions. In addition, interstitial I3 sites were identified. MEM analysis also revealed two orientations of the methylammonium ion, both by the positions of the C/N themselves, as well as by the hydrogen scattering density, where 4 distinct positions contained scattering from approximate three hydrogen atoms. The Pb, I, C/N and H positions were all confirmed with Rietveld refinement of the powder neutron diffraction pattern. Similar density maps for Pb, I, and C/N were obtained from MEM analysis of single crystal X-ray diffraction data.

2. To confirm the structure and obtain more accurate description of the iodide ions, single crystal X-ray diffraction was carried out. Refinement of the atomic coordinates confirmed the I2A and I3 positions, as well as the off-center C/N positions. All atomic coordinates referred to in the main text, as well as bond distances, are derived from the single crystal X-ray diffraction data at room temperature.

3. Given the additional positions and its implications on the iodide ion migration, we performed additional synchrotron powder X-ray diffraction experiments to monitor the values of the occupancies as a function of temperature. These are described in the main text.

Supplementary Note 2

To complement the three independent forms of diffraction that have been analyzed using both standard refinement methods as well as MEM to probe the local symmetry, we have carried out a series of Raman spectroscopy measurements. Supplementary Figure 6 shows the room temperature Raman spectra using the near infrared excitation wavelength of 784.15 nm. It is well established from previous Raman experiments[1] that Pb – I associated motions are observed around the 100 – 140 cm^{-1} region of the Raman spectra, whereas methylammonium vibrations are focused between 210 – 280 cm-^{1}. In addition to these two signatures, we observed further scattering at around 420 cm^{-1}. I_2 molecules confined within nanopores of a zeolite have previously been observed to give Raman signals at around 210 and 420 cm^{-1}, corresponding to the first two vibrational quantum numbers[2]. The first signal of the I_2 molecule at 210 cm^{-1} overlaps with the MA signal in the same frequency range. However, the secondary scattering at 420 cm^{-1} is consistent with confined I_2 molecules. Furthermore, other Raman studies of MAPbI have shown evidence for the presence of polyiodide species[1], which is assigned to signals at higher frequencies compared to established polyiodide studies[3].

Supplementary References

1 Pistor, P., Ruiz, A., Cabot, A. & Izquierdo-Roca, V. Advanced Raman Spectroscopy of Methylammonium Lead Iodide: Development of a Non-destructive Characterisation Methodology. *Sci Rep* **6**, 35973 (2016).

2 Guo, W., Wang, D., Hu, J., Tang, Z. K. & Du, S. Raman Spectroscopy of Iodine Molecules Trapped in Zeolite Crystals. *Applied Physics Letters* **98** (2011).

3 Mittag, H., Stegemann, H., Füllbier, H. & Irmer, G. Raman Spectroscopic Investigation of N-Alkylurotropinium Polyiodides. *J. Raman Spectroscopy* **20**, 255 (1989).
